# Activation of the aryl hydrocarbon receptor in inflammatory bowel disease: insights from gut microbiota

**DOI:** 10.3389/fcimb.2023.1279172

**Published:** 2023-10-24

**Authors:** Jun-Jie Hou, A-Huo Ma, Yue-Hua Qin

**Affiliations:** Department of Gastroenterology, Shaoxing People’s Hospital, Shaoxing, China

**Keywords:** inflammatory bowel disease, aryl hydrocarbon receptor, gut microbiota, interplay, therapy

## Abstract

Inflammatory bowel disease (IBD) is a chronic inflammatory intestinal disease that affects more than 3.5 million people, with rising prevalence. It deeply affects patients’ daily life, increasing the burden on patients, families, and society. Presently, the etiology of IBD remains incompletely clarified, while emerging evidence has demonstrated that altered gut microbiota and decreased aryl hydrocarbon receptor (AHR) activity are closely associated with IBD. Furthermore, microbial metabolites are capable of AHR activation as AHR ligands, while the AHR, in turn, affects the microbiota through various pathways. In light of the complex connection among gut microbiota, the AHR, and IBD, it is urgent to review the latest research progress in this field. In this review, we describe the role of gut microbiota and AHR activation in IBD and discussed the crosstalk between gut microbiota and the AHR in the context of IBD. Taken as a whole, we propose new therapeutic strategies targeting the AHR–microbiota axis for IBD, even for other related diseases caused by AHR-microbiota dysbiosis.

## 1 Introduction

As a gastrointestinal (GI) chronic, relapsing inflammatory disease, inflammatory bowel disease (IBD) can be divided into ulcerative colitis (UC) and Crohn’s disease (CD) according to clinical manifestations, endoscopic manifestations, and pathological results. It appears as abdominal pain, diarrhea, bloody stool, emaciation, and even extraintestinal symptoms (e.g., joint pain, and arthritis). It is characterized as relapsing and chronic, thus deeply affecting patients’ health, work, and daily life (Chang, 2020). In the late 20th century, IBD was considered a Western disease that mainly affected populations in North America and Europe, yet after entering the 21st century, the incidence and prevalence of IBD have risen worldwide, especially rapidly in the East (Mak et al., 2020). Although the etiology of IBD has not been fully elucidated, accumulating evidence revealed that the onset of IBD involves the complex interaction between genetic, environmental, microbial, and immune factors (Chang, 2020). Unfortunately, IBD remains incurable, which is greatly limited by the complex and unknown pathogenesis. Currently, the treatment for IBD includes the use of drugs and/or surgery. Regarding drug therapy, the suppression of the overactivated immunity and inflammation is the main drug action mechanism that non-steroidal drugs (NSAIDs), corticosteroids, immunosuppressants, and biological agents have been widely used for in the treatment of IBD (Nakase et al., 2021). However, the drug therapy of IBD is generally complicated, with obvious side effects, and poor tolerance which lead to about 15% of IBD patients worldwide being forced to undergo surgery each year (Le Berre et al., 2020) . Complex and limited therapeutic options further increase the physical and psychological burden of IBD patients instead.

Recently, increasing attention has been focused on the aryl hydrocarbon receptor (AHR) due to its role in the regulation of inflammation, immunity, intestinal barrier function, and intestinal microecology (Pernomian et al., 2020; Sun et al., 2020). The demonstration that the AHR modulates the differentiation and activity of Th1 and Th17 cell populations (Rodríguez-Sosa et al., 2005; Quintana et al., 2008; Quintana et al., 2012) further prompted us to examine the expression and role of the AHR in IBD. Reportedly, the AHR is highly expressed and highly activated in healthy intestinal epithelial cells (IECs) (de Vos et al., 2022) while lower levels of the AHR have been detected in IBD patients (Monteleone et al., 2011; Qiu and Zhou, 2013; Zhao et al., 2016). The pivotal role of the AHR in IBD has also been revealed by animal research (Furumatsu et al., 2011; Wang et al., 2018). Accordingly, AHR activity has been proposed as a potential therapeutic target for IBD. Several studies have confirmed that colitis can be alleviated by AHR ligands through activating the AHR (Hanieh, 2014; Lv et al., 2018a; Lv et al., 2018b; Yu et al., 2018; Marafini et al., 2019; Chen et al., 2020; Lin et al., 2023). However, most of the recognized exogenous synthetic AHR ligands have been associated with toxicity, such as 2,3,7,8-tetrachlorodibenzo-p-dioxin (TCDD) and polycyclic aromatic hydrocarbon (PAH) (Abudahab et al., 2023), which hamper the widespread application of AHR-ligand therapy and forces us to find non-toxic natural AHR ligands.

Compelling evidence has accumulated supporting the close association between gut microbiota and the development and maintenance of IBD (Lee and Chang, 2021). The human GI tract harbors an incredibly myriad microbiota that provide a platform for several biochemical metabolic reactions, which is considered a functional organ. Notably, an abundance of AHR ligands are accommodated in the GI tract, most of which are microbial metabolites (Pinto et al., 2023). The microbiota-derived AHR ligands include tryptophan metabolites, short-chain fatty acids (SCFAs), and phenolic microbial metabolites (Pernomian et al., 2020). In addition to the effects of the microbial community on AHR activity, the AHR could modulate the gut microbiota in turn (Pinto et al., 2023), that sufficient AHR ligands can rebalance the intestinal microecology and minimize mucosal inflammation to resist bacterial translocation (Esser, 2016). The mechanisms of AHR–microbiota interplay are multifactorial, including inflammatory response (Monteleone et al., 2013; Pernomian et al., 2020), immunologic balance (Monteleone et al., 2013; Pernomian et al., 2020), intestinal barrier integrity (Esser and Rannug, 2015), intestinal homeostasis (Qiu et al., 2012), and carcinogenesis (Murray et al., 2014).

In brief, accumulating evidence has demonstrated a crucial role for microbiota, as well as AHR activity in the pathogenesis of IBD. The research interest regarding the AHR–microbiota crosstalk in various diseases has been rising recently; the field has progressed rapidly and will undoubtedly continue to advance at a fast pace. In this review, we discuss the effects of gut microbiota, aryl hydrocarbon receptors, and their interactions on IBD depending on current evidence from clinical and animal experiments. More importantly, we aim to offer constructive strategies for the prevention and treatment of IBD via regulating the AHR–gut microbiota axis in the future.

## 2 Disturbance of gut microbiota in IBD

### 2.1 Characteristics of the microbiome in healthy intestines

The complex intestinal microecology, composed of trillions of diverse microorganisms that colonize the GI tract, is necessary for intestinal homeostasis (Arumugam et al., 2011). More than 1,000 microbial species residing in the GI tract have now been identified (Rajilić-Stojanović and de Vos, 2014). *Firmicutes*, *Bacteroidetes*, *Actinobacteria*, *Fusobacteria*, *Proteobacteria*, *Verrucomicrobia*, and *Cyanobacteria* are predominant divisions in the gut microenvironment, among which, *Firmicutes* and *Bacteroidetes* account for the majority of microbiota at the phyla level among healthy controls (HCs) (Gagliardi et al., 2018; Glassner et al., 2020). The gut microbiota is dynamic and widely involved in inflammation, immunity, metabolism, and other physiological processes, and exerts a significant influence on both the physical and mental health of individuals (Adak and Khan, 2019). With microbial qualitative or quantitative imbalance, namely, dysbiosis, various diseases can be triggered, such as cardiovascular diseases (Witkowski et al., 2020), autoimmune diseases (Christovich and Luo, 2022), gastrointestinal cancer (Meng et al., 2018), irritable bowel syndrome (IBS) (Canakis et al., 2020), and IBD (Lee and Chang, 2021).

### 2.2 Dysbiosis of gut microbiota in IBD

Analyses of the composition of gut microbiota in IBD patients observed differences in specific intestinal bacterial species compared with HCs (Glassner et al., 2020; Haneishi et al., 2023). The *Firmicutes*/*Bacteroidetes* (F/B) ratio is widely considered to maintain intestinal homeostasis, which is widely decreased in IBD patients (Stojanov et al., 2020; Ma et al., 2021). Briefly, although the results vary among multiple studies, the consensus is toward dysbiosis in IBD mainly being characterized by the decreased beneficial microorganisms with anti-inflammatory capacities (e.g. *Bacteroides*, *Bifidobacterium*, *Faecalibacterium prausnitzii*, *Suterella*, *Clostridium* groups IV and XIVa, and *Roseburia*) (Mondot et al., 2011; Morgan et al., 2012; Quévrain et al., 2016; Zhou et al., 2018; Pittayanon et al., 2020; Zhou et al., 2021) together with increased bacteria with pro-inflammatory effects, such as *Enterobacteriaceae* (*Escherichia coli* and *Fusobacterium* (Palmela et al., 2018; Mirsepasi-Lauridsen et al., 2019)), *Pasteurellaceae*, *Ruminococcus*, and *Veillonellaceae* (Pittayanon et al., 2020). *Helicobacter*, *Listeria*, *Klebsiella*, *Methanosphaera stadtmanae*, *Salmonella*, and *Yersinia* were also demonstrated related to the onset of IBD (Wang et al., 2014). The abundance of SCFA-producing bacteria that possess anti-inflammatory abilities is also observed to be reduced in IBD, which consequently exacerbates symptoms of IBD (Liu et al., 2021; Haneishi et al., 2023). Other non-bacterial microbiota, such as archaeome, mycobiome, virome, and eukaryotic parasites, also change in IBD (Matijašić et al., 2020). Further, the microbial community also differs in UC and CD patients (Qin et al., 2010).

In addition to the specific taxa alterations, IBD patients are also characterized by a decreased total number of species and low microbial diversity (Mentella et al., 2020; Ma et al., 2021); even the disease activity and severity of IBD are associated with these alterations in the gut microbiota (Stojanov et al., 2020). Multiple genetic studies (especially genome-wide association research) have proposed the association between several IBD-related gene polymorphisms and microbial responses (Jostins et al., 2012). These findings all confirm the pivotal role of dysbiosis of gut microbiota in the pathophysiology of IBD. Microbiota have thus emerged as a potential target for IBD treatment that attracts increasing attention.

## 3 The role of the aryl hydrocarbon receptor in IBD

### 3.1 The AHR signaling pathway

**Figure 1:**
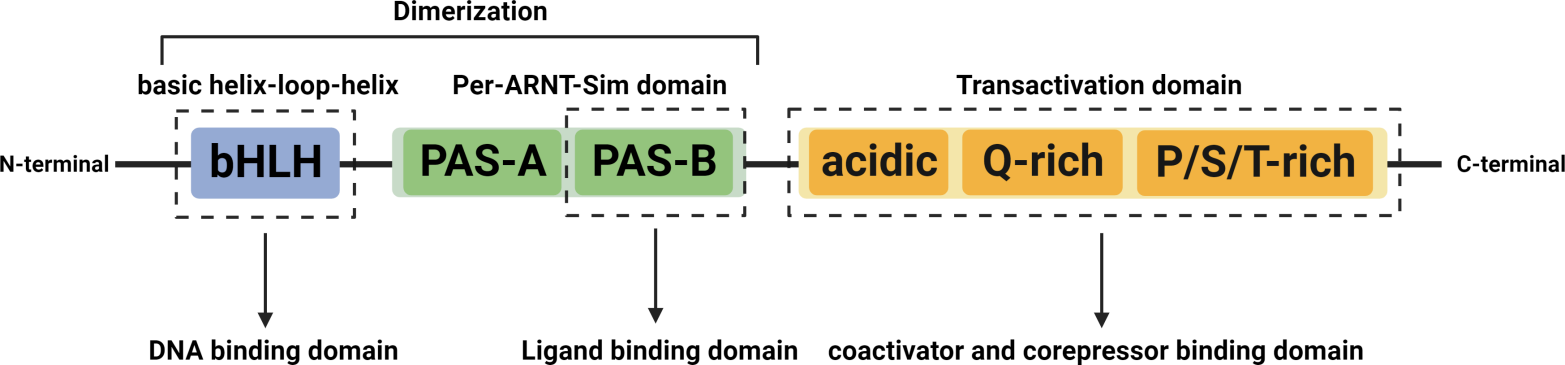
Structures of the AHR. The AHR contains three major domains, the bHLH, the PAS, and the TAD. The bHLH domain located at the N-terminus is involved in interaction with chaperones, HSP90, and XAP2, dimerization with ARNT, and binding to DNA. The PAS domain includes the PAS-A and PAS-B, among which, the PAS-A is involved in dimerization with ARNT and interaction with HSP90 and XAP2, while the PAS-B mediates AHR interaction with ligands. The C-terminal located TAD consists of three subdomains, an acidic residue (glutamate/aspartate) rich subdomain, a glutamate-rich subdomain (Q-rich), and a P/S/T region rich in proline/serine/threonine residues that are responsible for interaction with cofactors and mediating transcriptional activation. Abbreviation: AHR, aryl hydrocarbon receptor; ARNT, aryl hydrocarbon receptor nuclear translocator; bHLH, basic helix-loop-helix; TAD, transactivation domain; PAS, period circadian protein (PER) Aryl Hydrocarbon Receptor Nuclear Translocator | single-minded protein (SIM); HSP90, heat shock protein 90; XAP2, X-associated protein 2.

**Figure 2:**
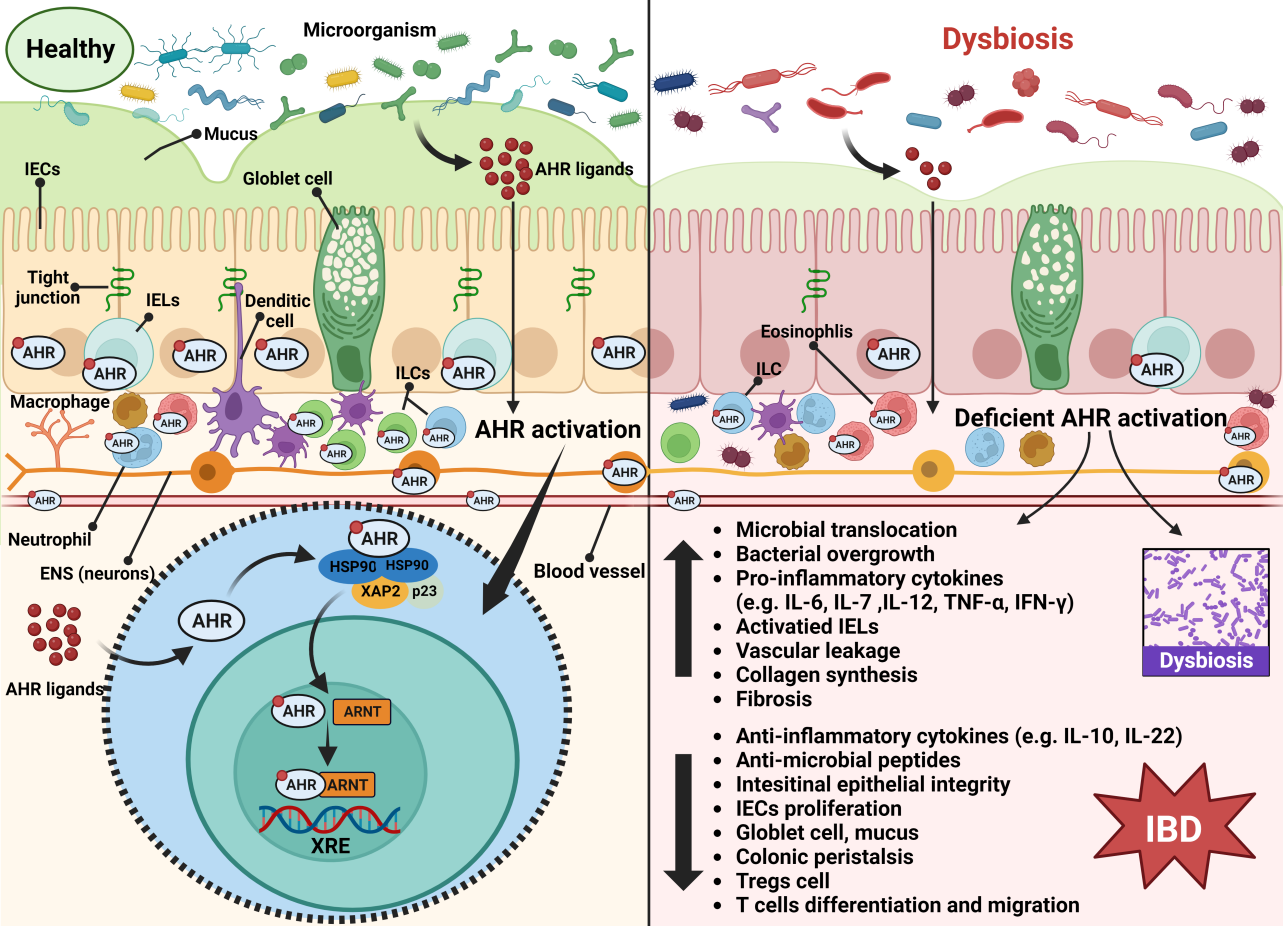
Summary of the possible mechanism of AHR activation in the onset of IBD under the context of dysbiosis. The AHR is expressed by epithelial cells, endothelial cells, immune cells, and neurons of the gut. Upon microbial AHR ligand triggering, AHR activity maintains intestinal homeostasis involving multi-factors, including modulation of intestinal barrier integrity, immune and inflammatory responses, intestinal peristalsis and vascular function, and gut microbiota. Once the intestinal microecology is disturbed, the deficient AHR activation will be triggered, which may lead to increased microbial translocation, bacterial overgrowth, pro-inflammatory cytokines production, IELs activation, vascular leakage, collagen synthesis, and fibrosis, and decreased production of anti-inflammatory cytokines, anti-microbial peptides, Treg cells, goblet cells and mucus, and intestinal barrier damage, along with restriction of IEC proliferation and T cell differentiation and migration. These explain the role of AHR activation in the pathogenesis of IBD. Abbreviation: AHR, aryl hydrocarbon receptor; ARNT; aryl hydrocarbon receptor nuclear translocator; ENS, enteric nervous system; HSP90, heat shock protein 90; IBD, Inflammatory bowel disease; IECs, intestinal epithelial cells; IELs, intraepithelial lymphocytes; ILCs, innate lymphoid cells; XAP2, X-associated protein 2; XRE, xenobiotic response element.

The AHR is a ligand-activated transcription factor, as well as a member of the basic helix-loop-helix/PER-ARNT-SIM (bHLH/PAS) family, ubiquitous in vertebrate cells (Kewley et al., 2004). It consists of an N-terminal bHLH domain, two PAS domains (PAS A and PAS B), and a C-terminal transactivation domain (TAD) where coactivators and corepressors interplay (**Figure 1**) (Stockinger et al., 2021). In the absence of ligands, the AHR resides in the cytoplasm in a transcriptionally inactive state as part of a complex with the chaperone proteins, known as two molecules of the heat shock protein 90 (HSP90), one molecule of the X-associated protein 2 (XAP2, also known as AIP), and one molecule of the HSP90 cochaperone p23 (Petrulis and Perdew, 2002). After ligand binding, the activated AHR translocates into the nucleus, where it dimerizes with its partner protein, the AHR nuclear translocator (ARNT), forming a functional transcription factor (Petrulis and Perdew, 2002). The AHR/ARNT complex then initiates the regulation of various target gene expressions by binding to the DNA of the entire genome, mainly the xenobiotic response element (XRE) (Quintana, 2013). The detailed process above can be seen in **Figure 2**.

The AHR can exert different effects by binding various exogenous and endogenous ligands with different structural and physicochemical properties (Denison and Nagy, 2003). However, the AHR activity is tightly controlled by three different negative feedback loops: (1) proteasomal degradation of the AHR induced by ubiquitin ligase complex; (2) metabolism clearance of ligands by Cytochrome P450 1A1 (CYP1A1); and (3) the interruption of the AHR/ARNT complex by the AHR repressor (AHRR) (Lamas et al., 2018b). In addition to these canonical signaling pathways, non-canonical mechanisms have also been revealed. During non-canonical signaling pathways, the AHR can: (1) interact with nuclear factor kappa-B (NF-κB), thus decreasing the expression of Cyp1A1 and increasing the expression of cytokines and chemokines including B-cell-activating factor of the tumor necrosis factor family (BAFF), B-lymphocyte chemoattractant (BLC), CC-chemokine ligand 1(CCL1), and interferon responsive factor (IFR3); (2) bind to Kruppel-like factor 6 (KLF6), which mediates cell cycle control; (3) combine hypophosphorylated retinoblastoma protein (pRb) to block cell cycle progression; and (4) interplay with the estrogen receptor (ER) and facilitate the proteolysis of the ER (Lamas et al., 2018b).

Overall, the ligand-activated AHR could regulate various gene expressions to maintain healthy cell physiology. Indeed, AHR ligands can be divided into exogenous (industrial synthesis and dietary), endogenous (indole and tryptophan metabolites), and other ligands (some chemicals, such as caffeine, nicotine, and pyridines) (Lamas et al., 2018b). Nowadays, searching for AHR ligands could be a novel strategy against IBD.

### 3.2 The alternation of the AHR in IBD

After a genome-wide linkage analysis of a large Danish family, Eiberg, H. et al (Eiberg et al., 2023). found that the AHR gene on the 7p-tel region was a candidate susceptibility gene for UC. Another study from Southeast China found an association between CD and AHR gene polymorphism in a group of patients that the genotypes of AHR (rs2158041) may be a CD susceptibility gene (especially for ileal CD and stricture CD) (Wu et al., 2019). These studies reasoned that the AHR gene may be the candidate susceptible gene for IBD. Consistently, diminished levels of AHR were observed in both colonic mucosa (Monteleone et al., 2011; Qiu and Zhou, 2013; Zhao et al., 2016) and fecal samples (Lamas et al., 2016) from IBD patients compared with HCs. Among these studies, the gene and protein expression levels of the AHR decreased markedly in the inflamed mucosa of CD patients compared with uninflamed tissues of HCs, but not in UC patients (Monteleone et al., 2011; Qiu and Zhou, 2013; Zhao et al., 2016). Additionally, decreased endogenous AHR ligand levels were reported in IBD patients (Hao et al., 2013; Esser, 2016; Lamas et al., 2016; Rothhammer et al., 2016). Monteleone, I. et al (Monteleone et al., 2011). observed no difference in terms of AHR activation between UC and HCs and suggested that the reduced AHR activation in CD is not an epiphenomenon of persistent inflammation. They also proved that the immune cells of IBD patients tend to express low AHR levels, especially in CD patients. Specifically, AHR expression was decreased in CD3^+^, CD4^+^, CD56^+^, and CD25+ cells through flow cytometry analysis of the LP mononuclear cells (Monteleone et al., 2011). Li, J et al (Li et al., 2016). subsequently made similar findings that the AHR expression in group 3 innate lymphoid cells (ILC3s) was lower in the inflamed intestinal biopsies of CD patients compared with unaffected tissues. Interestingly, different results have also been published. Arsenescu, R., et al (Arsenescu et al., 2011). reported a different result, finding that the colonic expression of the AHR was elevated in CD patients compared with HCs. The reasons for this discrepancy are unclear, which may partly be due to the different methods of AHR assessment and the smoking status of the study population in that the dioxins contained in cigarettes could induce the AHR.

Therefore, diminished AHR activity may be related to the onset and related symptoms of IBD, which has also been proved in animal studies. In murine models, AHR-deficient mice (AHR−/−) were more sensitive to dextran sodium sulfate (DSS)-induced colitis (Monteleone et al., 2011; Basu et al., 2012; Chinen et al., 2015; Schiering et al., 2017), showed higher levels of pro-inflammatory cytokines than wild-type (WT) (namely AHR+/+) controls, and more severe colitis-related symptoms and clinical outcomes (Takamura et al., 2010; Arsenescu et al., 2011; Furumatsu et al., 2011; Metidji et al., 2018; Wang et al., 2018). Accordingly, AHR agonists are capable of ameliorating colitis in mice (Cervantes-Barragan et al., 2017; Aoki et al., 2018), while AHR antagonists enhanced the severity of colitis (Monteleone et al., 2011). All these suggest that the AHR may play an important role in IBD, and the mechanisms involved in its protective role in maintaining intestinal homeostasis require more research.

### 3.3 The mechanism of low AHR activity for IBD

It has been reported that the AHR is highly expressed in healthy colon cells (mainly epithelial cells and immune cells in the lamina propria) (de Vos et al., 2022), while the loss of AHR activity is related to inflammatory intestinal disorders (Qiu and Zhou, 2013; Wu et al., 2019; de Vos et al., 2022). Here, we elaborate on the current insights into the mechanism of the AHR in intestinal inflammation, immunity, barrier, motility, and vascular homeostasis that may count for the initiation of IBD. The latent mechanism is shown in **Figure 2**.

#### 3.3.1 AHR in the intestinal barrier

Reportedly, AHR-deficient mice displayed impaired intestinal gut barriers (Di Meglio et al., 2014), which indicates the protective effects of the AHR in sustaining intestinal barrier integrity. The IECs form a physical and biochemical barrier as the first innate protection against pathogen invasion. The AHR is highly expressed in IECs (Stockinger et al., 2011; Stange and Veldhoen, 2013) and exerts barrier-protective effects after activation by natural ligands (Stockinger et al., 2021). Foremost, AHR signaling is necessary for local stem cells to differentiate into IECs (Metidji et al., 2018). Further, the AHR could control the bacterial load (Li et al., 2011) and is capable of goblet cells and mucus production (Yin et al., 2019) which, once lacking, can lead to bacterial translocation. Additionally, the AHR could modulate tight junction (TJ) proteins to maintain the intestinal barrier. The DSS-induced colitis mouse model is characterized by a significant loss of TJs proteins (ZO-1, claudin-1, and occludin), which could be markedly reversed by administration of 6-formylindolo [3,2-b] carbazole (FICZ) (Park et al., 2015; Yu et al., 2018), an endogenous AHR ligand (Rannug et al., 1995). Moreover, antioxidant urolithin A could main the gut barrier and function through the intersection of the AHR and nuclear factor erythroid 2-related factor 2 gene (Nrf2)/NF-κB pathways (Singh et al., 2019). IL-22, targeting IECs, has been shown to promote IECs proliferation (Han et al., 2022), induce antibacterial proteins, and enhance the mucus barrier (Sugimoto et al., 2008; Zenewicz et al., 2008). AHR activation ensures the production of IL-22 to exert a wide variety of beneficial effects on the intestinal barrier (Lee and Lee, 2010; Han et al., 2022). The AHR could also control the intraepithelial lymphocytes (IELs) which are associated with the gut barrier and inflammation development thus maintaining the barrier integrity (Li et al., 2011; Ji et al., 2015). Another mechanism by which the AHR protects the intestinal barrier is by reducing colonic inflammation. Consistently, AHR-deficient mice are more susceptible to pathogens (Kimura et al., 2008; Lee and Lee, 2010; Kiss et al., 2011; Lee et al., 2011). After the administration of AHR agonists, researchers observed that the activated AHR could offset the dysfunction of the intestinal barrier induced by tumor necrosis factor-a (TNF-a) and interferon-gamma (IFN-γ) (Yamada et al., 2016; Yu et al., 2018).

#### 3.3.2 AHR in inflammation

Apart from the improved intestinal permeability, researchers have also found reduced colonic inflammation (e.g., IL-6, IL-1β, TNF-α) and overall disease activity index (DAI) after urolithin A therapy which active the AHR in 2, 4, 6trinitrobenzenesulphonic acid (TNBS)-induced colitis models (Singh et al., 2019). The potential modulatory role of the AHR on intestinal inflammation may be through suppressing the expression of pro-inflammatory cytokines such as TNF-α, IFN-γ, IL-6, IL-12, IL-7, IL-1β, and IL-17 while promoting the expression of anti-inflammatory cytokines such as IL-10 and IL-22 (Qiu and Zhou, 2013; Hanieh, 2014; Wang et al., 2018; Pernomian et al., 2020). What is more, upon AHR triggering, the microbial translocation and collagen synthesis or fibrosis could be restricted by increased anti-microbial peptides (Pernomian et al., 2020). The prospective role of the AHR as an inhibitor of NOD-like receptor thermal protein domain associated protein 3 (NLRP3) inflammasome (linked to bowel inflammation) for IBD is also worth noting (Ngui et al., 2020). Of note, AHR activation induced high amounts of IL-22 produced by innate lymphoid cells (ILCs) and CD4^+^ T cells, which enhance the intestinal barrier and induce anti-microbial peptides, thus restricting bacterial translocation in dysbiosis and reduce intestinal inflammation (Marafini et al., 2019; Chen et al., 2020; Pernomian et al., 2020). Additionally, AHR activation may constrain T cell responses, contributing to inflammation control (Monteleone et al., 2011; Lamas et al., 2018b). Th17/Treg imbalance promotes the pathogenesis of IBD (Quintana et al., 2008). As such, AHR ligands, including TCDD (Singh et al., 2011) and 3, 3′-diindolylmethane (DIM) (Huang et al., 2013), could restore the Th17/Treg ratio to alleviate experimental colitis by inhibiting Th17 proliferation and inducing Treg differentiation. Meanwhile, Th17 cells-produced IL-17, which is involved in acute and chronic inflammation, could be negatively regulated by the activated AHR pathway (Hao et al., 2013; Chng et al., 2016; Ye et al., 2017). Takamura et al. (2010) proposed that the inhibition of colitis by the AHR may be due to the increased production of Prostaglandin E2 (PGE2) and that the inhibition of PGE2 reduced the inhibitory effects of the AHR on colitis. These confirmed the positive effect of AHR activation on intestinal inflammation. Consistently, the AHR agonist medication attenuates colitis in mice models (Benson and Shepherd, 2011; Monteleone et al., 2011; Huang et al., 2013).

#### 3.3.3 AHR in immunity

As expressed by different intestinal immune cells, including IELs, ILCs, Th17 cells, macrophages, dendritic cells (DCs), and neutrophils, the AHR is considered to participate in both innate and adaptive immune responses and regulates the immune homeostasis in the gut, which defends against infections (Qiu et al., 2013; Esser and Rannug, 2015; Lamas et al., 2018b). AHR ligands, including TCDD (Singh et al., 2011; Liu et al., 2020), FICZ (Qiu and Zhou, 2013; Hanieh, 2014; Wang et al., 2018), and Norisoboldine (NOR) (Lv et al., 2018a; Lv et al., 2018b), could ameliorate colitis symptoms by suppressing Th17 differentiation, leading to the increased IL-22 production, as well as decreased expression of IFN-γ and IL-17. NOR was also found to promote Treg cell differentiation by inhibiting Th1 differentiation (Tong et al., 2016; Lv et al., 2018b). Strikingly, the salient role of ligand-activated AHR in inducing tumor immune escape has been demonstrated by various studies (Ye et al., 2017; Cheong and Sun, 2018). Apart from the inhibited T cell responses by AHR activation (Kerkvliet et al., 1990), decreased lymphocyte accumulation in lymph nodes and the spleen (Fernandez-Salguero et al., 1995) and increased expression of pro-inflammatory cytokines (e.g., IL-12 and IFN-γ) in spleen cells (Rodríguez-Sosa et al., 2005) induced by AHR deficiency have also been reported. Kynurenine, another endogenous AHR ligand, is capable of regulating Treg cell functional maturation, combined with inhibiting inflammatory cytokine production thus altering the progression of inflammation and immunity balance (Mezrich et al., 2010; Díaz-Díaz et al., 2016). Another potent AHR agonist IPyA has exhibited remarkable anti-inflammatory effects in experimental colitis models through raised colonic IL10-positive T cells and lessening the pro-inflammatory Th1 cytokines (Aoki et al., 2018). Conversely, AHR antagonists elevated Th1 cytokines production and exacerbated the severity of TNBS-induced colitis in mice (Monteleone et al., 2011).

#### 3.3.4 AHR in intestinal motility

Surprisingly, the AHR expression and function in the enteric nervous system (ENS) were identified only 2 years ago (Stockinger et al., 2021). Adult mice studies showed that the sustained expression of the AHR in the ENS, which is restricted to colonic neurons, was found to be microbiota dependent (Obata et al., 2020). Serotonin, known as 5-hydroxytryptamine (5-HT), is in charge of GI motility, sensation, and secretion as a key neurotransmitter of the ENS (Spencer and Hu, 2020). Nevertheless, microbial metabolites promote 5-HT expression, in line with which, peristaltic defects were observed in germ-free mice due to the decreased 5-HT levels (Yano et al., 2015). However, by way of enhancing the induction of the AHR to Cyp1a1, 5-HT could influence the AHR activity in human IECs (Manzella et al., 2020). The AHR ENS neurons of mice showed delayed colonic transit, indicating colonic peristaltic activity is driven to some extent by ligand-dependent AHR activation (Obata et al., 2020). Obata, Y. et al. observed the expression of the AHR in the enteric neurons of mice treated with antibiotics partially restores intestinal motility (Obata et al., 2020). A previous dilated cardiomyopathy-related study showed that the promotion of peristalsis was partly attributed to AHR-mediated transcriptional upregulation of KCNJ12, a lipid-gated ion channel, which is responsible for encoding the primary subunit for IK1 (a key passage for cardiomyocytes). Recently, Zhizhu decoction has been proven to alleviate slow transit constipation by activating the AHR signaling pathway through gut microbiota (Wen et al., 2023). Interestingly, Vijay, A. et al. found that AHR activation affects nitrergic neuronal survival and delays intestinal motility in mice (Vijay et al., 2023). In brief, the AHR plays a regulatory role in intestinal motility, but the relevant mechanisms and studies are still limited and need to be further explored in the future.

#### 3.3.5 AHR in intestinal vascular homeostasis

Excessive angiogenesis is associated with the AHR in intestinal vascular homeostasis dysregulated repair responses, as a novel component of IBD pathogenesis (Pousa et al., 2008). Given the role of the AHR in IBD protection, along with growing evidence for the AHR in regulating vascular homeostasis, the function of the AHR in vascular remodeling and regeneration warrants further study. As reported, the involvement of laminar fluid shear stress represents a homeostatic function of AHR signaling in vascular tissue (Conway et al., 2009; Lano et al., 2020). The anti-inflammatory responses of AHR agonists in vascular tissue have been summarized previously (Bock, 2019). Recently, a study demonstrated for the first time that endothelial AHR ligand sensing is a key node in maintaining normal blood vessels of endothelial cell subtypes, thus regulating intestinal homeostasis (Wiggins et al., 2023). Further, they uncovered: 1) AHR ligands act directly on endothelial cells to promote quiescence and anti-inflammatory programs; 2) AHR facilitates vasculoprotective pathways in human endothelial cells (Wiggins et al., 2023). Although studies on the role of the AHR in the intestinal vasculature are limited, the AHR in endothelial cells has been presumed to potentially influence intestinal repair and regeneration upon injury, in which pro-inflammatory and angiogenic signals released by epithelial cells and immune cells at the injury site can trigger angiogenesis (Stockinger et al., 2021). Anti-inflammatory responses of AHR agonists in vascular tissue have been discussed in 2019 (Bock, 2019). In addition, the AHR in the vascular system is also involved in microbial defense (Stølevik et al., 2011).

Collectively, aside from the effect on the intestinal barrier, inflammation, immunity, motility, and vascular homeostasis, the AHR can also modulate the gut microbiota, which will be discussed in the next section.

## 4 AHR–Gut microbiota axis in IBD

The expression of the AHR and its target genes is lower in germ-free mice (Korecka et al., 2016). Also, caspase recruitment domain 9 (CARD9), one of the IBD susceptibility genes, links to AHR activation by interacting with the gut microbiota thus influencing the tryptophan (Trp) metabolism and IL-22 induction (Lamas et al., 2017). The microbiota from CARD9 knockout mice showed inhibited Trp metabolism, along with less production of AHR ligands IL-22, which eventually increased the risk of colitis (Rivas et al., 2011; Lamas et al., 2016; Lamas et al., 2017). Further, the microbiota of IBD patients with the risk allele of CARD9 also failed to adequately trigger the AHR activity, which may result in intestinal inflammation (Lamas et al., 2016). Hence, it is plausible to assume a relationship between the AHR and the gut microbiota in IBD.

### 4.1 AHR shapes the gut microbiota

As mentioned above, the AHR could control the bacterial load, induce anti-microbial peptides, and resist bacterial translocation (Takamura et al., 2010; Li et al., 2011; Esser, 2016; Marafini et al., 2019; Yin et al., 2019; Chen et al., 2020; Pernomian et al., 2020). Correspondingly, the diminished anti-microbial peptides induced by decreased AHR activity have been found rescued by FICZ treatment in mice (Wang et al., 2018). Through suppressing the neurogenic locus notch homolog protein 1 (Notch 1) signaling, the AHR can also produce more mucus to regulate the microbiome (Alvarado et al., 2019). Additionally, AHR expression has been shown to maintain specific ratios of various bacterial populations in the cecum, as well as modify the gut microbial community (Murray et al., 2016). For example, ILC3 from AHR-mediated IL-22 production was found to be critical in shaping the microbiota to mediate early colonization resistance to *C. rodentium* infection (Monteleone et al., 2011; Basu et al., 2012; Guo et al., 2015; Schiering et al., 2017). Furthermore, 2,3,7,8-tetrachlorodibenzofuran (TCDF) disrupted metabolism, altering the microbiota composition in mice by activating the AHR signaling (Zhang et al., 2015). Additionally, diets rich in AHR ligands influence the gut microbiota in mice. After feeding mice a diet lacking AHR ligands, researchers observed decreased abundance of *Bacteroidetes*, *Actinobacteria*, and *Tenericutes* in AHR-deficient mice, compared to WT mice (Korecka et al., 2016). They subsequently revealed that diets rich in AHR ligands upregulated the abundance of *Firmicutes* and downregulate the abundance of *Bacteroidetes*. The ability of the AHR to assess the relative abundance of bacterial communities has been described by several studies (Quintana et al., 2008; Kiss et al., 2011; Moura-Alves et al., 2014; Esser and Rannug, 2015; Moura-Alves et al., 2019). As a final note, delayed colonic transit induced by loss of the AHR in neurons leads to bacterial overgrowth in patients (Stockinger et al., 2021). Therefore, the AHR could modulate the gut microbiota (Pinto et al., 2023) and rebalance the intestinal microecology (Esser, 2016).

### 4.2 AHR modulation by intestinal microbiota

Both diminished levels of microbial AHR ligands and AHR activity were observed in fecal samples from IBD patients compared with HCs (Lamas et al., 2016). The role of the gut microbiota in providing AHR ligands has also been emphasized by many studies (Zelante et al., 2013; Hubbard et al., 2015; Lamas et al., 2016; Marinelli et al., 2019). Dong et al. (2020) observed alterations in AHR ligand production in germ-free mice. They identified both endogenous and microbiota-derived tryptophan metabolites present in mice cecal contents and human fecal samples, respectively, with the capacity to activate the AHR. These reveal the essential role of microbiota in AHR activity. Indeed, microbial derivatives including Trp metabolites and short-chain fatty acids (SCFAs) realized AHR activation. Precisely, Trp metabolites derived from microbiota represent an important source of endogenous AHR ligands (Sun et al., 2020), while SCFAs are essential for regulating the activation of the AHR induced by Trp-derived AHR ligands (Jin et al., 2017; Visekruna and Luu, 2021; Modoux et al., 2022). Several microbial Trp metabolites endowed with AHR agonist activity, including kynurenine, indole, serotonin, and tryptamine, are involved in the regulation of intestinal homeostasis (Zelante et al., 2013; Jin et al., 2014; Lamas et al., 2016; Liang et al., 2018; Kurata et al., 2019; Scott et al., 2020; Salminen, 2023). Moreover, a high-fat diet has been shown to alter the intestinal microbiome and its ability for metabolite production, such as tryptamine and indole-3-acetate (I3A), both of which are AHR agonists (Jin et al., 2014; Krishnan et al., 2018). Regarding microbiota-derived SCFAs, it not only increased the responsiveness and activation of the AHR (Jin et al., 2017; Marinelli et al., 2019) but also supported the growth of microorganisms with the capacity to metabolite Trp, which promotes AHR activity in B cells (Rosser et al., 2020). Reportedly, co-cultures of *Lactobacillus acidophilus* and *Bacillus subtilis* enhanced the mucosal barrier through bacterial SCFAs which upregulated the expression of the AHR and IL-22 (Xie et al., 2022). Gut microbiota-derived 3-phenylpropionic acid can also promote intestinal epithelial barrier function via AHR signaling (Hu et al., 2023).

To date, multiple bacteria species, including *Bacteroides fragilis*, *Bacteroides thetaiotaomicron*, *Citrobacter* sp (Chung et al., 1975)., *Clostridia* (Elsden et al., 1976), *Clostridioides difficile* (Lawson et al., 2016), *Clostridium sporogenes* (Dodd et al., 2017), and *Lactobacillus reuteri* (Natividad et al., 2018; Lamas et al., 2020; Bender et al., 2023) have been proved to produce the AHR agonist. Other microbial metabolites such as 2,8 dihydroxyquinoline have also been found to have capacity for AHR activation in human cells (Hubbard et al., 2019). Nevertheless, microorganisms may produce AHR antagonists, which could potentially affect AHR signaling locally and systemically in health and disease. A 2020 study examined the effect of 11 microbial tryptophan metabolites as AHR agonists/antagonists in detail (Vyhlídalová et al., 2020). Recently, both host and microbial-origin AHR ligands described as AHR agonists/antagonists have been summarized (Pinto et al., 2023), among which gut phenolic metabolite also exerts positive effects as an AHR modulator. Searching for AHR ligands could be a novel strategy against IBD (Pinto et al., 2023).

### 4.3 The AHR–microbiota axis in IBD

As discussed above, the AHR–microbiota axis in IBD has been brought to light. Both microbiota and AHR activity are deemed potential targets for IBD diagnosis and treatment. Nowadays, various therapies targeting the microbiome for IBD such as probiotics, antibiotics, fecal microbiota transplantation (FMT), defined enteral nutritional therapy (ENT), and gene manipulation have been discovered and demonstrated to have varying degrees of efficacy (Glassner et al., 2020). The positive effect of AHR agonists in colitis models has also been proved (Benson and Shepherd, 2011; Furumatsu et al., 2011; Monteleone et al., 2011; Singh et al., 2011; Huang et al., 2013; Qiu and Zhou, 2013; Hanieh, 2014; Ji et al., 2015; Park et al., 2015; Aoki et al., 2018; Lv et al., 2018a; Lv et al., 2018b; Wang et al., 2018; Yu et al., 2018; Marafini et al., 2019; Singh et al., 2019; Chen et al., 2020; Liu et al., 2020; Lin et al., 2023). However, both the markable immunotoxic effects and even carcinogenesis properties of most recognized exogenous synthetic AHR ligands have been elucidated (Murray et al., 2014; Stockinger et al., 2021; Abudahab et al., 2023; Chen et al., 2023), which limited clinical widespread application in IBD patients. Therefore, searching for non-toxic natural AHR ligands and exploring a series of potential mechanisms in the treatment of IBD has become a research hotspot in recent years.

Considering the AHR–microbiota axis of IBD, related therapies targeting the microbiota may achieve the remission of IBD targeting AHR activity. It has been proposed that insufficient microbial AHR agonists may contribute to the pathogenesis of IBD, metabolic syndrome, and other conditions (Lamas et al., 2016; Miani et al., 2018; Natividad et al., 2018). However, probiotics-produced AHR agonists have been successfully used to dampen inflammation in colitis. For instance, *Akkermansia muciniphila*, a promising probiotic, protects against colitis via activating AHR-Trp signaling (Gu et al., 2021a). Two *Bifidobacterium bifidum* strains, FL-276.1 and FL-228.1, were reported to ameliorate DSS-induced colitis by promoting the AHR pathway, which safeguards the barrier function (Cui et al., 2022; Cui et al., 2023). Similar results have also been reported in *Lactobacillus rhamnosus* (Luo et al., 2022; Niu et al., 2022), *Bifidobacterium breve* (Park et al., 2023), and co-cultures of *Lactobacillus acidophilus* and *Bacillus subtilis* (Xie et al., 2022). Additionally, *Lactobacillus reuteri* (Cervantes-Barragan et al., 2017; Özçam et al., 2019) and *Lactobacillus bulgaricus* strain OLL1181 (Perdew and Babbs, 1991; Takamura et al., 2010; Takamura et al., 2011) have been described as AHR activators both in mice models and human colonic samples. Other species including *Propionibacterium freudenreichii* ET-3, which is isolated from Swiss-type cheese, are also able to activate the AHR signaling, induced anti-microbial peptides, and improve DSS-induced colitis (Fukumoto et al., 2014). A recent study demonstrated three strains of *Lactobacillus* (*L. murinus*, *L. reuteri*, and *L. taiwanensis*) from feces of WT mice rescued impaired IL-22 production and high susceptibility to DSS of germ-free WT mice colonized by CARD9 deficiency microbiota (Lamas et al., 2016).

Beyond the probiotics, FMT also represents a novel therapy for IBD as an AHR promoter, yet is not fully clarified. After FMT from normal mice, the abundance of *Bifidobacterium* and *Lactobacillus* were augmented in colitis mice, along with the AHR expression and the levels of the anti-inflammatory cytokine, which revealed an association between the microorganisms and AHR activity, thus restoring the intestinal homeostasis and attenuated colitis (Wei et al., 2018). Withal, it is also possible to balance the gut microbiota through a diet rich in AHR ligands to relieve IBD patients (Daniel et al., 2014; Esser, 2016; Lamas et al., 2018a; Liang et al., 2018). After DSS gavage, both WT mice, which feed on a vegetable-free diet, and AHR-deficient mice displayed an increased abundance of *Bacteroides* and severe colitis (Li et al., 2011). However, these alterations can be attenuated by the adoption of vegetable-derived AHR ligands, which highlights the role of the AHR in the negative control of gut inflammation (Li et al., 2011). Aside from the reshaping of the gut microbiota, there are beneficial effects of long-lasting adherence to the Mediterranean diet for IBD patients also owing to the abundant specific components like phenolic metabolites, which are described as AHR agonists/antagonists thereby regulating AHR activity (Pinto et al., 2023).

**Figure 3:**
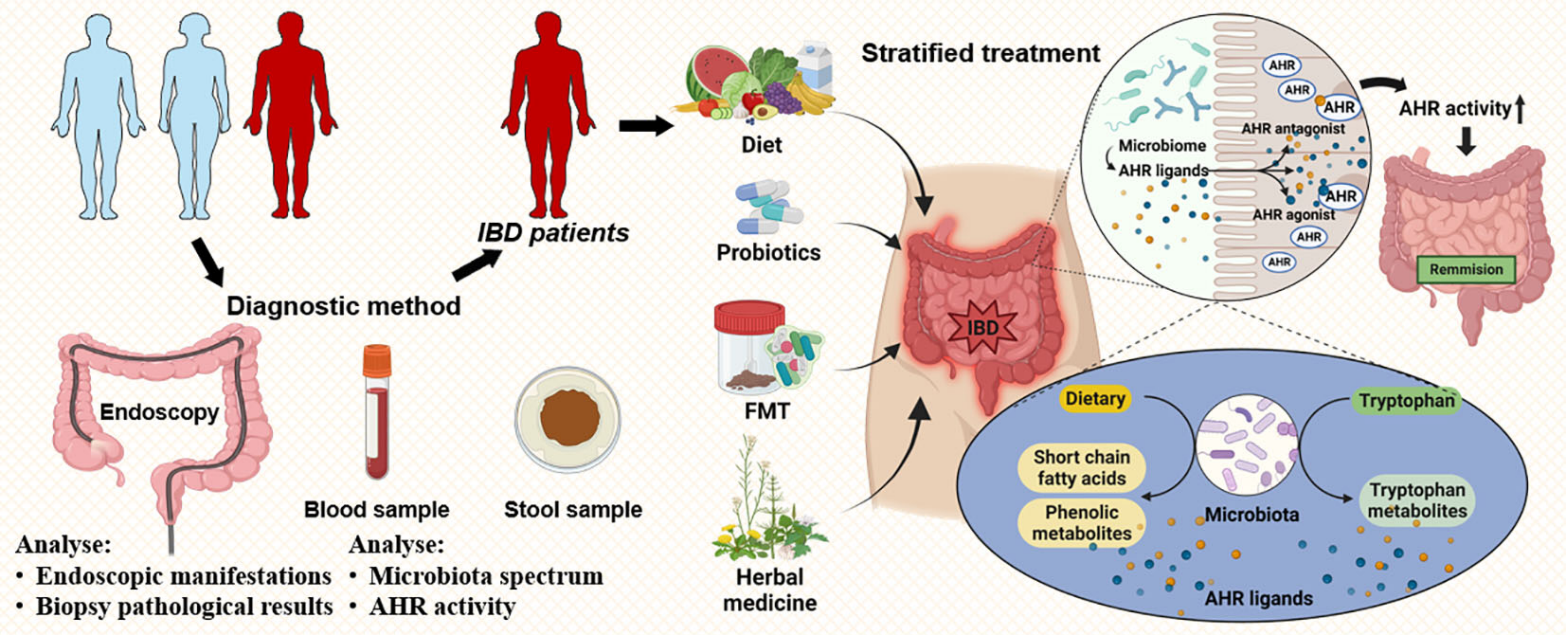
Prospects in IBD therapy targeting the AHR–gut microbiota axis. Due to the altered AHR activity and microbiome in IBD, the detection of AHR activity and the microbiome may become a new method for the diagnosis of IBD in addition to endoscopy. Further, the dysbiosis of IBD patients can be reconstituted by diet, probiotics, FMT, and herbal medicine. Notably, the microbiome produced the tryptophan metabolites, short-chain fatty acids, and other metabolites that act as AHR agonists or inhibitors to regulate AHR activity. Thus, a restored healthy intestinal microbiome may upregulate the AHR activity based on the AHR–gut microbiota axis, thus alleviating IBD. Abbreviation: AHR, aryl hydrocarbon receptor; IBD, Inflammatory bowel disease; FMT, fecal microbiota transplantation.

Additionally, herbal medicine has shown potential efficacy for IBD regarding the AHR-microbiota axis. Berberine (Jing et al., 2021), Shenling Baizhu San (Lv et al., 2022), Gegen Qinlian decoction (GQD) (Wang et al., 2023), and baicalein (Li et al., 2022) can greatly prevent the intestinal barrier and inflammation in the animal colitis model induced by DSS by regulating the AHR–microbiota axis. Recently, a random-controlled 8-week trial showed the efficacy of natural indigo, a Chinese herb containing AHR ligands, for UC patients (Naganuma et al., 2018). The therapies targeting the AHR activity for IBD in the context of microbiota is shown in **Figure 3**.

## 5 Future prospects

The great potential of the AHR–microbiota axis in IBD is well discussed in the previous section. However, the emergence of the AHR–microbiota axis prompted us to expand insights into other GI diseases, and AHR activity-related diseases including Alzheimer’s disease (Salminen, 2023), arthritis (Rosser et al., 2020), cardiovascular disease (Paeslack et al., 2022), psoriasis (Qiao et al., 2022), and so on. Consistently, the positive effect of *Lactobacillus rhamnosus GG* in alcohol-associated liver disease (ALD) was associated with its bacterial AHR ligand-enriched exosome-like nanoparticles (ELNPs), which increased expression of Reg3 and Nrf2 thereby improving barrier function (Gu et al., 2021b). Additionally, *Bacillus subtilis* KC1 enhanced AHR activation thus alleviating lung injury (Chen et al., 2023). *Bifidobacterium longum* CCFM1029 reshaped the gut microbial composition in atopic dermatitis patients, and activated the AHR-mediated immune response, alleviating related symptoms (Fang et al., 2022). Moreover, the AHR activated by *Lactobacillus reuteri* helps alleviate *Escherichia coli*-induced mastitis in mice (Zhao et al., 2021). In addition to probiotics, supplementation with dietary AHR ligands could alleviate *Escherichia coli*-induced endometritis in mice (Zhao et al., 2022). Withal, tapinarof, a bacteria-derived AHR agonist, has recently been indicated to resolve skin inflammation in mice and humans (Smith et al., 2017). Likewise, quinolinic acid, an AHR agonist derived from skin microbiota, negatively regulates NLRP3 inflammasome through the AHR-Trp pathway in Psoriasis (Qiao et al., 2022). Furthermore, FMT upregulated the abundance of *Lactobacillus* (FMT vs Con; 84.98% vs 66.94%), which activated the AHR, thus increasing the expression of CYP1A2 and IL-22, maintaining Th17/Treg balance and immune homeostasis, improving chicken growth performance (Ma et al., 2023).

Herein, we speculated that the appropriate application of the microbiota-target therapies including diets, probiotics, herb medicine, and even FMT, maintains the homeostasis of the AHR activity and alleviates, treats, and prevents other diseases that are not limited to IBD.

## 6 Conclusions

Generally, as the potential mechanisms of IBD, there has been a recent surge in research interest towards gut microbiota and AHR activity, such as the AHR–microbiota axis. However, current research regarding the AHR–microbiota axis in IBD is limited, which may become the next research target. Notably, we described an in-depth insight into the potential therapeutic strategies for IBD but need clinical studies to be confirmed in the future. More than such, it also requires deeper knowledge in this field to expound on the modulation of the AHR–microbiota axis in human physiological homeostasis and other diseases.

## Author contributions

J-JH: Conceptualization, Formal Analysis, Software, Visualization, Writing – original draft, Writing – review & editing. A-HM: Validation, Visualization, Writing – review & editing. Y-HQ: Conceptualization, Writing – review & editing.
